# Successful Ablation of Cavotricuspid Isthmus-dependent Atrial Flutter Guided by Contact Force Vector in a Patient After a Tricuspid Valve Replacement

**DOI:** 10.1016/s0972-6292(16)30820-8

**Published:** 2014-12-15

**Authors:** Eri Goto, Kohki Nakamura, Takehito Sasaki, Shigeto Naito

**Affiliations:** Division of Cardiology, Gunma Prefectural Cardiovascular Center, 3-12 Kameizumi-machi, Maebashi City, Gunma 371-0004, Japan

**Keywords:** Cavotricuspid isthmus-dependent atrial flutter, Contact force vector, Tricuspid valve replacement, Catheter ablation

## Abstract

A 46-year-old man after a tricuspid valve replacement due to traumatic severe tricuspid regurgitation developed cavotricuspid isthmus-dependent counterclockwise atrial flutter. During a linear ablation using a contact force-sensing irrigated ablation catheter, the flutter could be terminated by a radiofrequency application within a deep pouch just below the bioprosthetic tricuspid valve.

## Case Report

**Figure 1:**
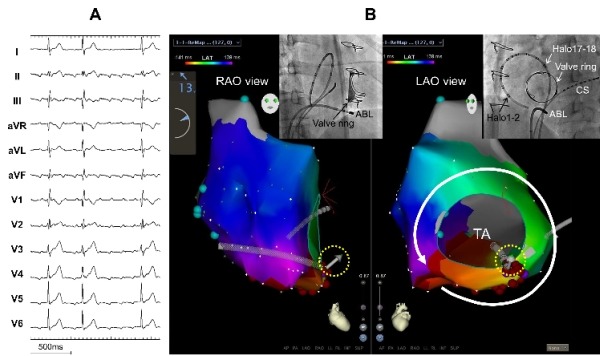
(A) Twelve-lead electrocardiogram during the clinicalAFL. (B) Activation maps of the AFL (white directional line) created by the CARTO^®^ 3 System and fluoroscopic images showing the position of an ablation catheter at the successful ablation site inthe right anterior oblique (RAO) and left anterior oblique (LAO) views. The CF vectors (yellow dotted circles) were directed towards the bioprostheticvalve within the pouch. ABL, ablation catheter; CS, coronary sinus; Halo 17-18 to 1-2, proximal to distal RA mapping catheter recordings; TA, tricuspid annulus.

A 46-year-old man after a tricuspid valve replacement (TVR) due to traumatic severe tricuspid regurgitation developed cavotricuspid isthmus (CTI)-dependent counterclockwise atrial flutter (AFL) (Figure 1). During a CTI linear ablation using a contact force (CF)-sensing irrigated ablation catheter (THERMOCOOL^®^ SMARTTOUCH™, Biosense Webster, Diamond Bar, CA, USA), the AFL could be terminated by a radiofrequency (RF) application within a deep pouch just below the bioprosthetic tricuspid valve, with a mean CF of 11g and CF vector directed towards the CTI. Bidirectional conduction block could not be completed despite repeated RF applications along the CTI. Finally, an ablation within the pouch with a mean CF of 17g and CF vector directed towards the valve, that is, an inverted vector with respect to the CTI (Figure 1, Panel B), completed the bidirectional CTI conduction block, and no AFL could be further induced. The patient remained free from any AFL recurrence during a one year follow-up without antiarrhythmic drugs.

## Discussion

**Figure 2:**
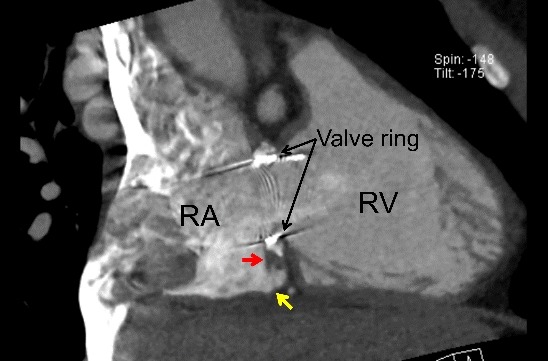
Double oblique computed tomographic image showing the pouch just below the bioprosthetic valve (yellow arrow) and atrial myocardium attached to the valve ring (red arrow). RA, right atrium; RV, right ventricle.

To the best of our knowledge, this is the first case describing the utility of a CF vector during a CTI linear ablation after a TVR. CTI pouches are usually considered to be major obstacles for creating linear RF lesions along the CTI. Furthermore, RF catheter ablation of a CTI-dependent AFL after a TVR has rarely been reported, [1-3] but is a challenging procedure due to the artificial valve-related risks. In this case, a deep pouch was close to the bioprosthetic valve ring and made it difficult to create complete conduction block along the CTI. This report suggested that the atrial myocardium attached to the valve ring served as a critical conduction pathway of the AFL (Figure 2), and the direction of the CF vector was of particular benefit when ablating within the pouch associated with a TVR and completing bidirectional CTI conduction block.
